# Immunohistochemical Expression of VEGF and Podoplanin in Uterine Cervical Squamous Intraepithelial Lesions

**DOI:** 10.1155/2016/8293196

**Published:** 2016-05-23

**Authors:** Patrícia Napoli Belfort-Mattos, Gustavo Rubino de Azevedo Focchi, Julisa Chamorro Lascasas Ribalta, Tatiana Megale De Lima, Carmen Regina Nogueira Carvalho, Fernanda Kesselring Tso, Neila Maria De Góis Speck

**Affiliations:** ^1^Gynecological Disease Prevention Nucleus (NUPREV), Department of Gynecology, Federal University of São Paulo, UNIFESP/EPM, 380 Borges Lagoa Street, 04038-000 São Paulo, SP, Brazil; ^2^Department of Pathology, Federal University of São Paulo, UNIFESP/EPM, 740 Botucatu Street, 04023-062 São Paulo, SP, Brazil

## Abstract

VEGF and podoplanin (PDPN) have been identified as angiogenesis and/or lymphangiogenesis regulators and might be essential to restrict tumor growth, progression, and metastasis. In the present study, we evaluate the association between the expression of these markers and CIN grade. Immunohistochemistry was performed in 234 uterine cervical samples using conventional histologic sections or TMA with the monoclonal antibodies to VEGF (C-1 clone) and podoplanin (D2-40 clone). Positive-staining rates of VEGF in 191 CIN specimens were significantly associated with histological grade (*P* < 0.001). Negative and/or focal immunostaining for PDPN were more frequent in CIN 3 (*P* = 0.016). We found that patients with CIN 3 more frequently had strong and more diffuse staining for VEGF and diminished staining for PDPN (*P* = 0.018). Strong and more diffuse VEGF immunoexpressions in CIN 2 and CIN 3 were detected when compared to CIN 1. Negative and/or focal PDPN immunoexpression appear to be more frequent in CIN 3. Moderate to strong VEGF expression may be a tendency among patients with high-grade lesions and diminished PDPN expression.

## 1. Introduction

Angiogenesis and lymphangiogenesis consist in the formation of new blood vessels and lymphatic vessels, respectively [1]. A great amount of signal transduction systems is involved in these processes [2], which are crucial for tumor growth, progression, and metastasis [3]. The upregulation of proangiogenic factors, such as vascular endothelial growth factor (VEGF), also known as VEGF-A, has been reported as the most important developmental regulators in this mechanism [4, 5]. This agent also supports lymphangiogenesis through interaction with VEGFR-2 expressed on lymphatic endothelial cells (LEC). VEGF induces proliferation of LEC and its overexpression* in vivo* induces lymphangiogenesis in tissue repair and inflammation [6, 7]. Studies revealed that VEGF also might stimulate lymphangiogenesis indirectly by recruitment of VEGF-C/-D secreting mononuclear cells [7–9]. The indirect effect of VEGF by increasing blood vessel formation promotes expansion of the pool of VEGF-C responsive blood endothelial cells that serve as precursors for early LEC differentiation [10].

Podoplanin (PDPN) acts in the separation of blood and lymphatic vasculature during angiogenesis [11]. It is exclusively detectable in lymphatic but not blood vessel endothelium [12] and has been widely used as a specific marker for lymphatic endothelial cells and lymphangiogenesis in many species of tumors [13, 14]. Its expression is upregulated in different types of cancers, including squamous cell carcinoma of oral cavity, the lung, head, and neck [15, 16], and, on the other hand, has not been found in the majority of adenocarcinomas, including lung, prostate, and colon [13]. In addition, it has been shown that low levels of PDPN expression in tumor cells were significantly associated with the presence of lymphatic invasion, lymph node metastasis, and shorter recurrence-free survival, but not with disease-related overall survival [17]. Although the biological function of PDPN is not fully understood [18], many studies have investigated the crucial role of PDPN expression in human cancers with the aim of employing PDPN expression as a prognostic marker [13].

Studies have reported findings in favor of the assumption that the process of lymphangiogenesis is present from the very beginning of the development of cancer and coexists with the process of angiogenesis [3]. Therefore, the expression of VEGF and PDPN in precancerous lesions might be essential to modulate or restrict lesion progression and tumoral metastatic dissemination. The aim of the present study was to evaluate and correlate the immunohistochemical expression of VEGF and PDPN in cervical epithelial cells of different degrees of squamous intraepithelial neoplasia (CIN) and also in nonneoplastic cervical tissue.

## 2. Patients and Methods

### 2.1. Patients

The study was approved through the Research Ethics Committee of the Federal University of São Paulo UNIFESP/EPM under protocol number 0337/11, and all participants previously agreed with and signed an informed consent form. This retrospective cross-sectional study included 234 cervical biopsies (191 with squamous intraepithelial neoplasia and 43 without neoplasia), which were selected from Gynecological Disease Prevention Nucleus between 2008 and 2015. Patients were diagnosed by colposcopic and histopathological studies, and the absence of neoplasia was confirmed through the cytological, colposcopic, and histopathological exam. Women with any kind of immunosuppression were excluded from the study. Cervical samples were distributed into CIN 1, CIN 2, CIN 3, and control (nonneoplastic) groups.

### 2.2. Clinical Method

Clinical data were collected from patients charts. All patients were submitted to anamneses, general physical, gynecological, and specular examinations for the collection of cervicovaginal cytology and colposcopy, with biopsy of any abnormal findings.

### 2.3. Conventional Histopathological Method

The paraffin embedded formalin fixed tissue blocks were cut into 4 *μ*m thick sections using an AO American optical 820 Rotary microtome (AO Instrument Company, New York, USA). Briefly, after assembly into a glass slide, the tissue was deparaffinized in xylene, rehydrated in graded alcohols, and submitted to hematoxylin and eosin (HE) staining followed by sealing with Entellan® (Merck Millipore, Darmstadt, GE). HE stained slides of all cases were reviewed and the diagnoses confirmed.

The HE stained slides of all cases were reviewed independently by two different experienced pathologists and the discordant diagnoses were resolved by consensus.

### 2.4. Construction of Tissue Microarray (TMA)

The construction of TMA was performed according to the standard technique previously described [19, 20]. Briefly, using corresponding HE slides as a guide, cylinders of 0.8 mm diameter were punched from selected areas of each donor paraffin block and these were mounted into a receptor paraffin block at 1 mm intervals using a precision microarray instrument (Beecher Instruments, Silver Spring, MD, USA). A grid system was previously established so each core would have a coordinate reference as *x*-axis and *y*-axis for sample identification. Blocks were sealed at 60° for 10 min before cutting of 4 *μ*m sections and prepared using standard methods. The sections were fixed into silanized slides and three selected glass slides were submitted to HE staining for evaluation of the samples before immunohistochemistry.

### 2.5. Immunostaining for VEGF and PDPN

Immunohistochemical staining was standardized to histological specimens for VEGF and PDPN through the immunoperoxidase activity. The glass slides were previously incubated with Pierce*™* APTS (3-aminopropyltriethoxysilane) (Thermo Fisher Scientific, Massachusetts, USA) at an oven at 60°C for 24 hours. Sections were dewaxed, rehydrated, and treated for quenching of endogenous peroxidase activity. To enhance the antigen retrieval, we used PT-link buffer (Dako, Denmark) with pH 9.0 at 97°C for 2 hours. Sections were washed with PBS 10 mM with pH 7.4 for 5 min, followed by incubation with specific antibody to VEGF and PDPN.

#### 2.5.1. VEGF-A

Sections were incubated with a mouse monoclonal antibody to VEGF at a dilution of 1 : 50 (C-1 clone, raised against amino acids of VEGF-A of human origin; sc-7269; Santa Cruz Biotechnology, CA, USA), followed by immersion in wash buffer for 5 min and amplification with polymer EnVision Flex*™* (Dako) for 20 minutes.

#### 2.5.2. PDPN

Sections were incubated with a mouse monoclonal antibody to podoplanin (D2-40 clone, prediluted, ready for use, M3619; Dako) for 20 min followed by immersion in wash buffer for 5 min and amplification with polymer EnVision Flex (Dako) for 20 minutes.

After incubation with the two antibodies, the samples were washed with buffer for 5 min and amplified with Novolink Novocastra Kit (Leica Biosystems, Nußloch, GE). Sections were washed with buffer, which contains the chromogen 3,3′-diaminobenzidine-tetrahydrochloride-dihydrate (DAB) (Sigma-Aldrich Chemical, St. Louis, Missouri, USA), 600 *μ*L hydrogen peroxide (V30), 100 mL of PBS, and 1 mL dimethyl sulfoxide, for 5 min at 37°C. Finally, the sections were counterstained with Harris hematoxylin and were mounted with Entellan (Merck Millipore, Darmstadt, GE).

We used placental tissue as an external positive control for both VEGF and PDPN. Blood vessel endothelium was used as an internal positive control for VEGF and lymphatic endothelium as an internal positive control for PDPN. Two pathologists carried out the evaluation independently.

VEGF expression was classified according to intensity of immunostaining in the cytoplasm and cytoplasmic membranes of epithelial cells: negative to weak, moderate, and strong. PDPN expression was identified using the criteria described by Han et al. [21].

### 2.6. Statistical Analysis

Analyses were performed using Excel 2010 for Windows and the R statistical package (2.15.2 version; Rio de Janeiro, Brazil). For a comparison of quantitative variables among the groups the ANOVA and Tukey tests were used, and the qualitative variables were performed by Chi-square tests and/or the exact Fisher tests. Statistical significance was established as *P* < 0.05.

## 3. Results

This study comprised 43 women in the control group (mean age 33.8 ± 9.3), 53 in the CIN 1 group (mean age 32.2 ± 9.1), 60 in the CIN 2 group (mean age 30.9 ± 7.5), and 78 in the CIN 3 group (mean age 36.0 ± 8.4), with ages ranging from 18 to 59 years old.

**Figure 1:**
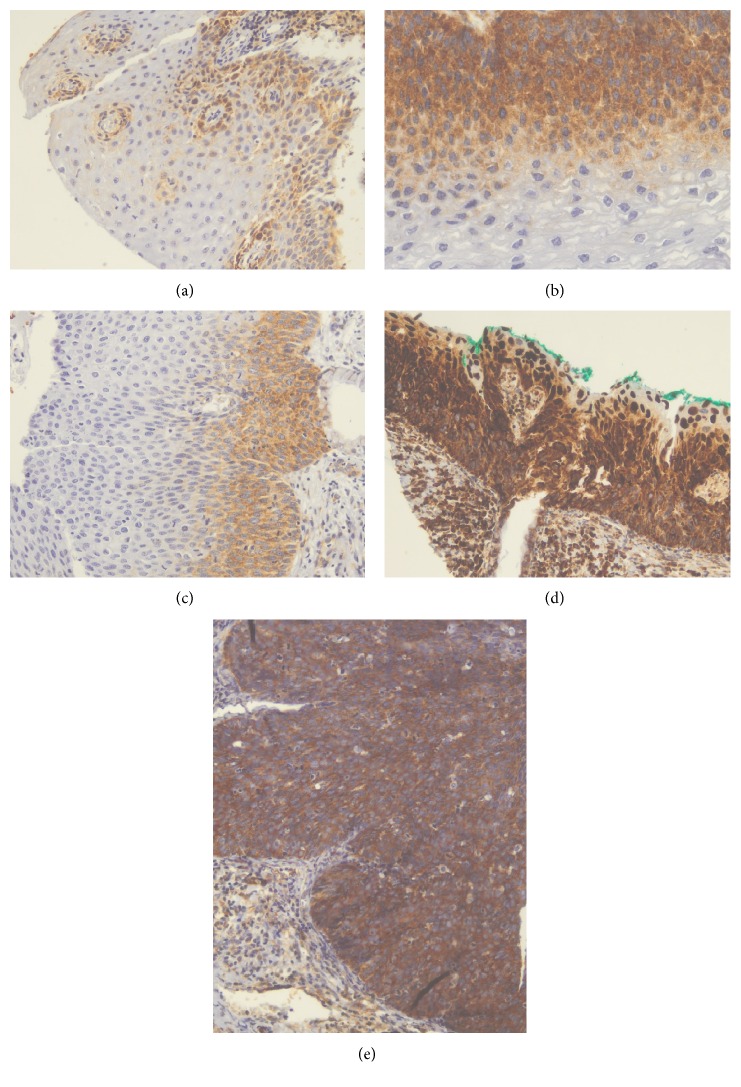
Microphotographs showing the immunohistochemical expression of VEGF in cervical tissues. (a) Nonneoplastic squamous epithelium with weak expression (×100), (b) CIN 1 with moderate expression (×200), (c) CIN 2 with moderate expression (×100), (d) CIN 2 with strong expression (×100), and (e) CIN 3 with strong expression (×200).

VEGF immunoexpression was detected the cytoplasm (Figure 1) and the comparison of staining intensity between the different groups showed a statistically significant difference (*P* < 0.001). There was no similarity in the occurrence of strong VEGF immunoexpression, which was more often detected in CIN 2 and CIN 3 groups (25% and 48.7%, resp.) when compared to CIN 1 (18.9%) and to the control group (4.7%) (Table 1). Intraepithelial lesions showed a progressive increase of moderate and strong VEGF expression according to the degree of neoplasia, which was noted in 35.85% of CIN 1, 60% of CIN 2, and 67.95% of CIN 3.

**Figure 2:**
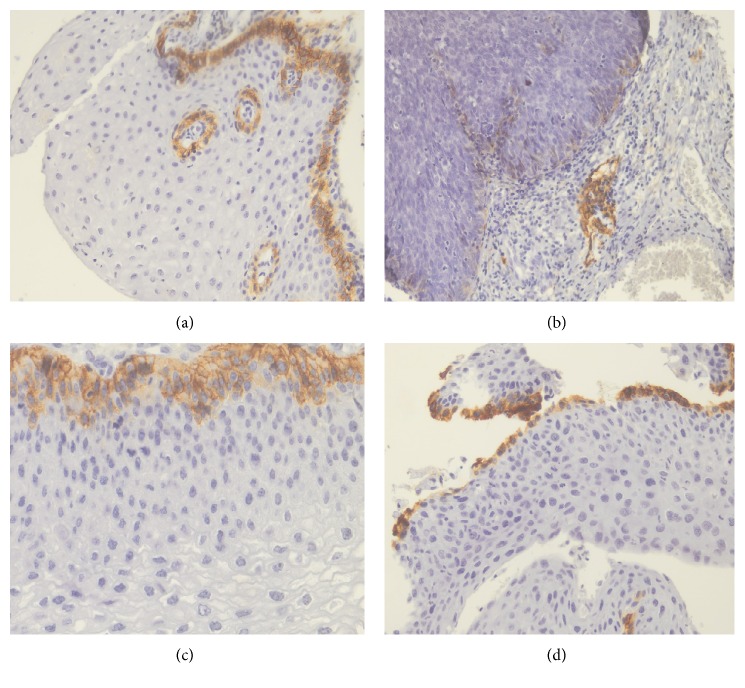
Microphotographs showing the immunohistochemical expression of PDPN in cervical tissues. (a) Nonneoplastic squamous epithelium showing diffuse expression in the basal layer (×100), (b) CIN 3 with negative/focal expression (×100), (c) CIN 1 with diffuse expression (×200), and (d) CIN 2 with diffuse expression (×100).

Negative or focal PDPN immunoexpression (Figure 2) was more frequent in the CIN 3 group when compared with the CIN 1, CIN 2, and control groups: only 61.5% of CIN 3 samples showed diffuse expression. In the comparisons between the different groups (Table 2), there was a statistically significant difference (*P* = 0.016).

Our data revealed that the groups did not necessarily present similar profiles when correlating VEGF and PDPN immunoexpression. Although the correlation of expression of VEGF and PDPN showed that both had similar frequencies in the control, CIN 1, and CIN 2 groups (Table 3), CIN 3 group demonstrated higher frequency of strong VEGF immunoexpression combined with negative/focal PDPN expression (60.0%) when compared to strong VEGF immunoexpression combined with diffuse PDPN expression (41.7%) (*P* = 0.018). In contrast to negative/weak VEGF immunoexpression (13.3%), moderate/strong VEGF immunoexpression was highly frequent (86.7%) in the negative/focal PDPN immunoexpression samples, compared to over half (56.25%) of moderate/strong VEGF immunoexpression in the samples with diffuse PDPN immunoexpression.

## 4. Discussion 

Proangiogenic agent VEGF is essential for vasculogenesis [22, 23], hematopoiesis, wound healing, and development [24]. Vascular proliferation is a feature of cervical cancers; high levels of VEGF expression are seen in these tumors [25] and high density of microvessels indicates a worse prognosis [26]. Previous immunohistochemical studies have shown a correlation between an increase of VEGF expression and cancer stage [27–30]. In our study, VEGF expression seems to play an important role in CIN 3, of which 67.9% of the samples showed moderate to strong immunoexpression. On the other hand, CIN 1 showed strong VEGF immunoexpression in only 18.9% of cases. Angiogenesis is highly associated with high-grade intraepithelial lesions [31]; thus, a correlation can be noted between the high expression of VEGF and potentially premalignant lesions. In addition, the metabolic remodeling and angiogenic switch are relevant to cancer progression and aggressiveness in adenocarcinomas [32].

Our results showed considerable differences in negative or weak VEGF immunoexpression between CIN 1 and CIN 3. In CIN 2 negative or weak expressions were present in 40% of the samples. Moderate or weak immunoexpressions were observed in about 51.2% of patients with CIN 3. We noted the occurrence of a progressive increase of strong VEGF expression in CIN 1 to CIN 2 to CIN 3 (18.9%, 25%, and 48.7% of the samples, resp.). This finding corroborates a previous study performed by us [33] that showed a progressive increase of VEGF immunoexpression in high-grade squamous intraepithelial lesions when compared to low-grade squamous intraepithelial lesions and to benign squamous epithelium. The progression of the carcinogenic process in the cervix was related to increasing VEGF immunoexpression. VEGF expression may stimulate tumor cell proliferation in the early stages of cervical cancer and may be responsible for cervical tumorigenesis [34].

In the current study, negative or weak VEGF immunoexpression was observed in 60.5% of the samples of the control group. Of note, moderate to strong VEGF immunoexpression was observed in almost 40% of the nonneoplastic samples. VEGF has also been shown to be expressed in another squamous epithelium under inflammatory conditions [35]. Our results corroborate the observation that inflammatory conditions can result in an increase of VEGF immunoexpression.

Transmembrane protein PDPN is expressed in endothelial cells of lymphatic vessels and it has been used as a lymphangiogenesis marker in solid tumors [13]. In our study, positive immunohistochemical reaction was observed in the lymphatic vessels and in the keratinocytes of the basal layer of normal or pathologic cervical squamous epithelium, as described in the literature [21, 36].

Our immunohistochemical analysis revealed that the frequency of diffuse PDPN expression was lower in CIN 3 (61% of samples) when compared to the other groups. Notably, the CIN 3 group did not present any PDPN expression in 6.4% of the cases and 7.7% exhibited very low expression. Notwithstanding, there were no samples with absent or very low expression in the CIN 1 group; 3.3% of CIN 2 samples had very low expression and none had absent expression. Curiously, the decrease in PDPN immunoexpression might be related to histological severity of cervical neoplasia and perhaps; it may indicate the tendency of more aggressive lesion development. Han et al. [21] observed moderate to strong expression of D2-40 antibody in normal cervical tissue and a similar pattern of immunoexpression was showed in CIN 1. Lower expression was found in CIN 2 and CIN 3, and a statistically significant difference was observed among CIN 1, CIN 2, and CIN 3. The reduction of PDPN expression is related to degree of aggression in the epithelium caused by precursor lesions, showing significant difference between low-grade and high-grade intraepithelial neoplasia. Longatto-Filho et al. [37] have shown that the lymphatic neovascularization begins early in intraepithelial lesions and continues to increase towards malignancy, and both lymphatic invasion and decrease in D2-40 expression in tumor cells appear to have a prognostic value. Moreover, the positivity in tumor cells was associated with a better prognosis in adenosquamous carcinoma in their study.

Lymphovascular invasion is a risk factor highly correlated with tumor recurrence in cervical squamous cell carcinoma. An association between low levels of PDPN in the tumor cells with the presence of lymphatic invasion and lymph node metastasis was found [17], suggesting that low expression of PDPN might be a risk marker of tumor recurrence and invasion of lymphatic system. Carvalho et al. [19] investigated the PDPN expression in the intratumoral stroma and neoplastic cells of early stage uterine cervical cancer. The findings showed that this marker may have a role in host-tumor interaction and, as a result, may represent a favorable prognostic factor for squamous cervical cancer. In addition, lymphatic vessels were observed both within the tumor mass and in the peritumoral area in other cancer types [30].

PDPN and VEGF immunoexpressions were simultaneously compared in the same fragments of low- and high-grade squamous intraepithelial lesions, but we did not find any statistical significance. The results to D2-40 antibody were equally frequent in the several types of VEGF in the control group. CIN 3 presented moderate to strong VEGF expression more frequent among the patients with negative and focal PDPN in 86.7% of the samples when compared to patients with diffuse PDPN expression (56.25%). The increase in VEGF immunoexpression was observed according to the histological severity of the precursor lesion and low expression of PDPN was observed in CIN 3 in comparison to CIN 1. We noted that VEGF expression increases in parallel with the histological severity of the cervical squamous intraepithelial lesion, confirming the hypothesis that VEGF might be of use as a prognostic remission marker and therapeutic response marker. Metastatic tumor spread through the blood or lymphatic vessels occurs in most forms of cancer, with regional lymph node metastasis often being the most important prognostic factor for carcinoma patients [29]. We found reduced PDPN expression in 38.5% of samples with CIN 3, giving us the idea that these subgroups of CIN 3 lesions may have a more aggressive/less regressive behavior.

Although the two studies factors are directly linked to the processes of lymphatic vasculature, it is essential to highlight that high-grade intraepithelial lesions may develop invasive neoplasia easily. Moreover, it is important to take into account that other functional alterations may occur during lesion development, explaining cases that progress to invasive cancer or relapses posttreatment.

Histological assessment is crucial to treatment of patients with precursor lesions; the medical follow-up would show how they evolved and the behavior of premalignant lesions. New biological markers are necessary to more precisely direct diagnosis and treatment of patients with cervical lesions, especially young women which often required cold knife conization, LETTZ, LEEP, or hysterectomy. Due to lack of consistent results about these biological markers in cervical lesions, we noticed the importance to evaluate the profile of VEGF and PDPN immunoexpression and show their relevance as predictor for cancer progression, which have a great potential to prevent metastasis development.

## 5. Conclusions

In summary, we found that the CIN 2 and CIN 3 groups seem to not demonstrate similarity in the occurrence of strong VEGF immunoexpression in comparison to the CIN 1 and control groups. Negative or focal PDPN expression was apparently more frequent in CIN 3 than in the other groups. Patients with CIN 3 appear to exhibit a tendency towards stronger VEGF expression and reduced PDPN expression. VEGF expression was markedly stronger in high-grade lesions than in low-grade lesions, which could be a possible screening tool. The results presented herein provide additional evidence that the simultaneous evaluation of VEGF and PDPN immunoexpression could add information to the treatment strategy in patients with CIN. Nonetheless, more studies are necessary to further our understanding about these markers in cervical cancer development and metastasis, since increasingly younger women are getting the disease by HPV infection.

## Figures and Tables

**Table 1:** Distribution of VEGF immunoexpression in squamous intraepithelial lesions.

VEGF	Control		CIN 1		CIN 2		CIN 3		Total		*P* value^*∗*^
	*n*	%	*n*	%	*n*	%	*n*	%	*n*	%	
Negative or weak	26	60.5	34	64.2	24	40.0	25	32.1	109	46.6	<0.001
Moderate	15	34.9	9	17.0	21	35.0	15	19.2	60	25.6	
Strong	2	4.7	10	18.9	15	25.0	38	48.7	65	27.8	
Total	43	100	53	100	60	100	78	100	234	100	

^*∗*^Chi-square test.

**Table 2:** Distribution of PDPN immunoexpression in squamous intraepithelial lesions.

PDPN	Control		CIN 1		CIN 2		CIN 3		Total		*P* value^*∗*^
	*n*	%	*n*	%	*n*	%	*n*	%	*n*	%	
Negative or focal	9	20.9	9	17.0	12	20.0	30	38.5	60	25.6	0.016
Diffuse	34	79.1	44	83.0	48	80.0	48	61.5	174	74.4	
Total	43	100	53	100	60	100	78	100	234	100	

^*∗*^Chi-square test.

**Table 3:** Correlation between VEGF and PDPN immunoexpression in squamous intraepithelial lesions.

Diagnosis	VEGF	PDPN				Total		*P* value
		Negative or focal		Diffuse				
		*n*	%	*n*	%	*n*	%	
Control	Negative or weak	7	77.8	19	55.9	26	60.5	0.147^a^
	Moderate	1	11.1	14	41.2	15	34.9	
	Strong	1	11.1	1	2.9	2	4.7	
	Total	9	100	34	100	43	100	

CIN 1	Negative or weak	4	44.4	30	68.2	34	64.2	0.252^a^
	Moderate	3	33.3	6	13.6	9	17.0	
	Strong	2	22.2	8	18.2	10	18.9	
	Total	9	100	44	100	53	100	

CIN 2	Negative or weak	2	16.7	22	45.8	24	40.0	0.110^a^
	Moderate	7	58.3	14	29.2	21	35.0	
	Strong	3	25.0	12	25.0	15	25.0	
	Total	12	100	48	100	60	100	

CIN 3	Negative or weak	4	13.3	21	43.8	25	32.1	0.018^b^
	Moderate	8	26.7	7	14.6	15	19.2	
	Strong	18	60.0	20	41.7	38	48.7	
	Total	30	100	48	100	78	100	

^a^Fisher's exact test; ^b^Chi-square test.
